# A Plant-Derived Recombinant Human Glucocerebrosidase Enzyme—A Preclinical and Phase I Investigation

**DOI:** 10.1371/journal.pone.0004792

**Published:** 2009-03-11

**Authors:** David Aviezer, Einat Brill-Almon, Yoseph Shaaltiel, Sharon Hashmueli, Daniel Bartfeld, Sarah Mizrachi, Yael Liberman, Arnold Freeman, Ari Zimran, Eithan Galun

**Affiliations:** 1 Protalix Biotherapeutics, Science Park, Carmiel, Israel; 2 Goldyne Savad Institute of Gene Therapy, Hadassah-Hebrew University Hospital, Jerusalem, Israel; 3 Gaucher Clinic, Shaare Zedek Medical Center, Jerusalem, Israel; Instituto Butantan, Brazil

## Abstract

Gaucher disease is a progressive lysosomal storage disorder caused by the deficiency of glucocerebrosidase leading to the dysfunction in multiple organ systems. Intravenous enzyme replacement is the accepted standard of treatment. In the current report, we evaluate the safety and pharmacokinetics of a novel human recombinant glucocerebrosidase enzyme expressed in transformed plant cells (prGCD), administered to primates and human subjects. Short term (28 days) and long term (9 months) repeated injections with a standard dose of 60 Units/kg and a high dose of 300 Units/kg were administered to monkeys (n = 4/sex/dose). Neither clinical drug-related adverse effects nor neutralizing antibodies were detected in the animals. In a phase I clinical trial, six healthy volunteers were treated by intravenous infusions with escalating single doses of prGCD. Doses of up to 60 Units/kg were administered at weekly intervals. prGCD infusions were very well tolerated. Anti-prGCD antibodies were not detected. The pharmacokinetic profile of the prGCD revealed a prolonged half-life compared to imiglucerase, the commercial enzyme that is manufactured in a costly mammalian cell system. These studies demonstrate the safety and lack of immunogenicity of prGCD. Following these encouraging results, a pivotal phase III clinical trial for prGCD was FDA approved and is currently ongoing.

**Trial Registration:**

ClinicalTrials.gov NCT00258778

## Introduction

Since its introduction in 1991, glucocerebrosidase enzyme replacement therapy (ERT) has become the standard of care for patients with symptomatic Gaucher disease due to its safety and efficacy profile [1]–[4]. The success of ERT in Gaucher disease ultimately led to the development of recombinant enzyme treatments for other lysosomal storage diseases such as Fabry, MPS-I, MPS-II, MPS-IV, Pompe and other lysosomal storage disorders [5]–[11]. Currently, the enzymes used for treating lysosomal storage disorders in general and in Gauchers disease in particular are expressed in mammalian, Chinese Hamster ovary cells (CHO)[1]. However, production of this enzyme in mammalian cells is expensive, and the high cost of the approved recombinant glucocerebrosidase for treating Gaucher's disease, is raising public concern [3], [12], [13].

In an attempt to offer an alternative source for the production of the glucocerebrosidase enzyme, we have developed a biotechnological expression platform which is based on the industrial scale expression of human recombinant proteins in genetically engineered plant cells [14]. The plant cell technology allows for a cost efficient production system. In addition, the entire manufacturing process is free from any animal-derived components, complementing processing safety advantages as well. prGCD, is the most clinically advanced recombinant plant system expressed protein to undergo phase III clinical trials [15] and its chemical, functional and genetic characterization, including the full amino-acid sequence and its three dimensional crystal structure have recently been described [14]. Following the successful completion of non-clinical safety toxicology studies, which included a single dose study in rodents [14] and a 28-day acute safety toxicology study in primates (Cynomolgus monkeys) with daily dosing of prGCD, regulatory approval for conducting a Phase I clinical trial was allowed by the FDA. The clinical study in healthy human volunteers was designed to evaluate the safety of three escalating doses of prGCD and to determine the pharmacokinetics profile of the drug. In addition, a nine-month chronic safety toxicology study in primates (Cynomolgus monkeys) with dosing once every two weeks, mimicking the proposed clinical regime of prGCD, was also performed. This study was a prerequisite for the initiation of an advanced Phase III clinical trial, which will address the continuing safety of chronic administration of prGCD.

## Materials and Methods

The protocol for this trial and supporting CONSORT checklist are available as supporting information; see Checklist S1 and Protocol S1.

### Non clinical safety studies in Cynomolgus Monkeys

Two extensive safety toxicology studies were performed: an acute 4-week daily intravenous infusion study and a chronic 39-week intravenous infusion study in Cynomolgus Monkeys. In each study, twenty four (24) animals (4/sex/dose) were intravenously infused either daily (in the acute study) or once every 2 weeks (in the chronic study) over 1 hr with multiples of 1 or 5 times the clinical dose (60 units/kg) adjusted to animal body surface area. The doses of 5.6 and 27.8 mg/kg/day represent 1× and 5× the clinical dose on a mg/m^2^ basis, respectively. The clinical dose of 60 units/kg, equivalent to approximately 1.8 mg/kg in humans, corresponds to 66 mg/m^2^ (using the conversion factor of 37 kg/m^2^ for humans), and corresponds to 5.6 mg/kg in cynomolgus monkeys (using the conversion factor of 12 kg/m^2^ for Cynomolgus monkeys). Studies were performed according to Good Laboratory Practice (GLP) at MPI Research (Mattawan, Michigan, USA). This facility maintains an Animal Welfare Assurance statement with National Institutes of Health, Office of Laboratory Animal Welfare. All experiments were performed in accordance with the guidelines of the Animal Care and Use Committee of the Hebrew University. Animals were subjected to clinical observations and assessed for body weight, hematology, coagulation, clinical chemistry and urinalysis evaluations. Animals were sacrificed at the end of treatment period and organ weights, macroscopic and microscopic pathology were also determined. Antibodies were measured on Day 1 and Day 28 in the acute study and on Day 1, followed by 1, 3, 6 and 9 months in the chronic study. Plasma concentrations and toxicokinetics were measured on Day 1 and Day 28 in the acute study and on Day 1, Week 9 and Week 39 in the chronic study.

### Phase I clinical trial in healthy volunteers

The phase I study was designed as a single-center, non-randomized, open label, safety and pharmacokinetic study of escalating single doses of prGCD administered as intravenous infusion (IV) to six healthy volunteers. This study was conducted according to FDA and European GCP guidelines, and was approved by the IRB of the Hadassah University Hospital and the Israeli Ministry of Health and under an FDA investigational new drug application (IND). The study was performed at the Phase I Unit at the Goldyne Savad Institute of Gene Therapy at the Hadassah Hebrew University Hospital. The study was approved by the Helsinki committee of Hadassah-Hebrew University Hospital Jerusalem, Israel, and all subjects gave written informed consent. Inclusion and exclusion criteria are presented in Table 1. Further information can be found at ClinicalTrials.gov website, ClinicalTrials.gov Identifier: NCT00258778 (the study initiated 22^nd^ of Nov 2005 and was completed 20^th^ of March 2006).

**Table 1:** Phase 1 study: Inclusion and Exclusion criteria.

Inclusion Criteria	Exclusion criteria
Healthy male or female between 18 and 45 years of age	Clinical evidence of any active significant disease that could potentially compromise the ability of the investigator to evaluate or interpret the effects of the study treatment on safety assessment and thus increase the risk to the subject to unacceptable levels
Female subjects must agree to use a medically acceptable method of contraception at all times during the study and must have a negative serum pregnancy test at baseline and during the study period.	Are pregnant or nursing
Females of child-bearing potential must be non-pregnant and not lactating and using adequate birth control such as oral contraceptives	Presence of any acute or chronic diseases
Negative laboratory tests for HIV, HBsAg or HCV	Have a history of any allergies
Naive to any previous recombinant protein therapy	Have been exposed to long-term steroid treatment
Provide written informed consent	Had a minor operation in the last 6 months
Have the ability to understand the requirements of the study and to comply with the study protocol and dosing regimen	Have ever been exposed to any previous recombinant protein therapy
	Have received immuno-suppressive treatment
	Have a positive HIV, HBsAg and HCV laboratory result
	Use any medication other than vitamins or oral contraceptives (for female).
	Have participated in another clinical trial during the previous 3 months
	Have a history of alcohol or drug abuse
	Are considered by the Investigator to be unsuitable candidate for this study.

### Study Protocol

A vehicle control placebo followed by three single escalating doses of prGCD were administered via intravenous (IV) infusions. The vehicle was administered at the baseline visit, followed by an initial prGCD dose of 15 units/kg administered on Day 8; 30 units/kg on Day 15, and 60 units/kg administered on Day 22. The infusion rate was 1.5 mL/minute (135 mL over 90 minutes), which was the same for all doses. Subjects were closely monitored for 8 hours from the time of the initiation of the infusion and returned after 24 hours from the time of initiation of infusion for blood sampling and additional safety assessments. A follow up visit was performed on day 29, at the end of the study. The issue of safety was the primary outcome in this study; safety measurements included adverse events, general infusion related toxicities, physical examinations including changes in vital signs and body weight, and laboratory tests. Pharmacokinetic parameters were the secondary outcomes in this study. Blood samples were collected prior to dosing (0) and at 5, 45, 80 and 90 minutes during the infusion and 100, 115, 130, 150, 180, 210 minutes and 24 hours from initiation of infusion, and the plasma was analyzed to provide a pharmacokinetic profile (AUC_last_, T_max_, C_max_ and C_min_).

The Pharmacokinetic analysis of the clinical samples was performed by Midwest Bioresearch LLC, Skokie, IL, USA.

### Statistical methods

Safety parameters at each follow-up assessment, as well as changes from baseline, were examined and summarized for descriptive purposes. Safety was also assessed through the recorded adverse events. Adverse events were classified by body system using the Medical Dictionary for Regulatory Activities (MedDRA), and tabulated based on the incidence, severity, and causality to study treatment.

Statistical analysis of the clinical trial data was performed by TechnoStat Ltd., Kfar Saba, Israel.

### Screening of anti-prGCD antibodies

Analysis of anti-prGCD antibodies was performed using a validated immunoassay we have developed (Midwest BioResearch, LLC, Skokie, Illinois). Controls and unknown samples were incubated in a 96-well microtiter plate coated with prGCD molecules and incubated with shaking at room temperature, allowing any anti-prGCD antibodies present to bind to the prGCD molecules. After incubation, the plate was washed to remove any nonreactive serum components. Biotinylated prGCD conjugate was added which enabled binding to anti-prGCD antibodies already bound to the prGCD solid phase. Detection was performed using a streptavidin-peroxidase conjugate. The plate was washed to remove any unbound protein and reagents followed by the addition of the substrate, tetramethylbenzidine (TMB) solution. After a 10-minute incubation, 2 M sulfuric acid was added and the absorbance was measured at 450 nm. The intensity of the color produced was proportional to the concentration of anti-prGCD antibodies in the sample. Antibody-positive samples were determined by comparing the optical density (OD) with a predetermined cutoff OD. An OD equal to or greater than the cutoff identifies a sample as antibody-positive. Samples that were presumed positive underwent immunodepletion testing by addition of prGCD. The addition of prGCD to samples containing anti-prGCD was expected to block the anti-prGCD antibodies from binding to the prGCD coat on the plate, thereby decreasing the OD readings. Any sample demonstrating a decrease of greater than 50%, after the addition of prGCD, was considered positive for anti-prGCD antibodies.

### Determination of neutralizing antibodies

Analysis of neutralizing antibodies was performed (Midwest BioResearch, LLC, Skokie, Illinois) using a validated immunoassay we have developed. Controls and unknown samples were incubated in a 96-well microtiter plate coated with prGCD molecules and incubated with shaking at room temperature, allowing any anti-prGCD antibodies present to bind to the prGCD. After one hour of incubation at room temperature, the plate was washed to remove any non-reactive serum components. The substrate, p-nitrophenyl β-D-glucopyranoside, was mixed with the activity assay buffer and added to the wells. After a one-hour incubation at room temperature, 5 M sodium hydroxide was added to stop the reaction and enhance the intensity of the color developed. Control samples included neutralizing and non-neutralizing controls. A solution of prGCD, substrate and enzyme inhibitor conduritol B epoxide (CBE) which was added to the uncoated wells was used to perform enzyme inhibition. The control of pooled naive human serum was added to the coated wells to track the activity of the enzyme coated to the surface of the microtiter wells in the presence of normal human serum. A sample is considered positive for neutralizing activity if the OD obtained is less than or equal to the assay cutoff.

### Determination of prGCD concentration in serum

Determination of prGCD plasma concentrations was performed (Midwest BioResearch, LLC, Skokie, Illinois) using a validated immunoassay we have developed. Controls and unknown samples were incubated in a 96-well microtiter plate coated with chicken-anti-plant recombinant glucocerebrosidase (prGCD) antibodies and incubated with shaking at room temperature allowing any prGCD present to bind to the anti-prGCD antibodies. After two hours of incubation, the plate was washed to remove any non-reactive plasma components. Rabbit anti-prGCD antibodies were added and the plates were incubated 1.5 hours at room temperature to allow the rabbit anti-prGCD to bind to the prGCD. The plate was washed to remove any unbound protein and reagents followed by the addition of an alkaline phosphatase affinity-purified goat anti-rabbit IgG conjugate. After a 60-minute incubation at room temperature, the plate was washed and detection was performed with phosphatase substrate. The absorbance was measured at 405 nm and color was allowed to develop at room temperature for 30 to 45 minutes or until the optical density (OD) was 1.0 or greater. The intensity of the color produced was proportional to the concentration of prGCD in the sample. The prGCD levels were quantified according to a standard curve generated by measuring purified recombinant prGCD in a 4% cynomolgus monkey plasma or human plasma matrix utilizing a four-parameter curve fit equation.

### Pharmacokinetic and Toxicokinetics Calculations

Plasma analysis data were analyzed by Noncompartmental pharmacokinetic analyses (NCA) using WinNonlin®, version 5.0.1 software (Pharsight Inc., Mountain View, CA) and Microsoft Office Excel 2003. Plasma prGCD concentration versus time data for individual animals and human subjects were analyzed for maximum concentration (Cmax), the time at which Cmax occurred (Tmax), and for area under the plasma concentration versus time curve from the start of infusion to 24 hours post-infusion, using the linear trapezoid rule (AUCall). All AUC calculations were based on the time interval from start of infusion to the last measurable plasma concentration (AUClast). , Clearance (CL) and the volume of distribution at steady-state (Vss) were determined.

## Results

### Safety and toxicology studies in primates

No treatment-related side effects on survival or any clinical signs were observed during both the acute and chronic studies in primates. There was no effect of prGCD on electrocardiogram parameters, ophthalmological, hematological, coagulation, clinical chemistry, or urinalysis values. Organ weights were not affected and there were no investigational drug-related macroscopic or histopathology findings. All changes were considered to be within normal limits for cynomolgus monkeys. Based on these data, a no-observed-adverse-effect-level (NOAEL) of 27.8 mg/kg/day, the highest dose tested, was identified.

In the acute study, repeated daily dosing of monkeys with prGCD for 28 consecutive days did not result in production of any anti-prGCD antibodies or neutralizing antibodies. In the chronic study, samples from 5 of the 24 animals were found positive for anti-prGCD antibodies, 3 out of 8 animals receiving 5.6 mg/kg/day and in 2 out of 8 animals receiving 27.8 mg/kg/day. All antibodies were determined to be negative for neutralizing antibody activity.

All animals treated with prGCD had significant plasma exposure to the investigational drug. The prGCD plasma exposure profiles were similar at both dose levels. Plasma concentrations of prGCD increased with increasing dose of prGCD.

### Phase I Clinical Trial

Seventeen volunteers were assessed for eligibility. Six volunteers were enrolled and allocated for the study. All six had completed all study steps and their data was available for analysis. None had dropped out during the study period. The volunteers, 3 males and 3 females, were all Caucasian aged 19–36.

Six healthy volunteers were administered with the vehicle at the baseline visit, followed by an initial prGCD dose of 15 units/kg administered on Day 8; 30 units/kg on Day 15, and 60 units/kg on Day 22. Safety results indicated that prGCD was well tolerated with only non-drug- related, minor side effects that were self limited and resolved with no treatment. No serious adverse events were attributable to prGCD administered intravenously once a week at a dose of up to 60 units/kg. Laboratory tests for kidney and liver function tests were all within normal range. One volunteer had measurements of Bilirubin up to1.4 mg% (normal values 0.3–1 mg%) during the follow-up period and a second volunteer had measurements of Bilirubin up to 1.1 during the follow-up period ; all the rest had normal Bilirubin levels within normal limit all along the study.. Immunological laboratory tests were all within normal limits. The Complete Blood Counts (CBC) differentials revealed that one volunteer had at screening and at follow-up a slight elevation in eosinophils, up to 7% (normal values up to 5%; and his total WBC count was approximately 8,000/cm3); and the second volunteer had a single measurement of 6% of eosinophils (at a total WBC of 6,000/cm3). Complement Component 3 (C3) measurements: 5 measurements were found to be slightly lower than normal range. One subject had lower values at screening and at Visit 1, which were normal afterwards. One subject had three values lower than normal: at Visit 3, Visit 4 and follow-up. None of these results were clinically significant. IgE measurements were within normal range in five volunteers and only one exhibited a slightly high value, which was similar to his baseline and the vehicle control dosing period. Adverse events were classified by body system using MedDRA, and tabulated based on the incidence, severity, and causality to study treatment (Table 2). Administration of prGCD did not result in the formation of anti prGCD antibodies in any of the patients. Overall, prGCD presents a high safety profile.

**Table 2:** Adverse events classified by body system using MedDRA, based on the incidence, severity, and causality to study treatment

Relation between Event to drug dose and severity	Placebo	15 U/kg	30 U/kg	60 U/kg	Event Severity	Total # of events
**Unrelated to drug (1)**	0; 0 (0%)	0; 0 (0%)	0; 0 (0%)	2; 1 (17%)	Moderate	2
**Remotely related to drug (2)**	1; 1 (17%)	4; 2 (33%)	1; 1 (17%)	2; 1 (17%)	Mild	8
**Possibly related to drug (3)**	0; 0 (0%)	0; 0 (0%)	0; 0 (0%)	0; 0 (0%)	__	0
**Probably related to drug (4)**	0; 0 (0%)	0; 0 (0%)	0; 0 (0%)	0; 0 (0%)	__	0
**Definitely related to drug (5)**	0; 0 (0%)	0; 0 (0%)	0; 0 (0%)	0; 0 (0%)	__	0

(1) **The event is clearly related to other factors**, such as a subject’s clinical state, therapeutic interventions or concomitant medications.

(2) **The event was most likely produced by other factors**, such as a subject’s clinical state, therapeutic interventions or concomitant medications, and does not follow a known response pattern to the study drug.

(3) **The event has a reasonable temporal relationship to the study drug** administration and follows a known response pattern to the study drug. However, a potential alternate etiology may be responsible for the event. The effect of drug withdrawal is unclear. Re-challenge information is unclear or lacking.

(4) **The event follows a reasonable temporal sequence from the time of drug** administration, and follows a known response pattern to the study drug and cannot be reasonably explained by non drug related factors. There is a reasonable response to withdrawal of the drug. Re-challenge information is not available or advisable.

(5) **The event follows a temporal sequence from the time of drug administration and follows a known response pattern to the study drug**. Either occurs immediately following the study drug administration, improves on stopping the drug, or reappears on repeat exposure.

### Pharmacokinetic profile

**Figure 1:**
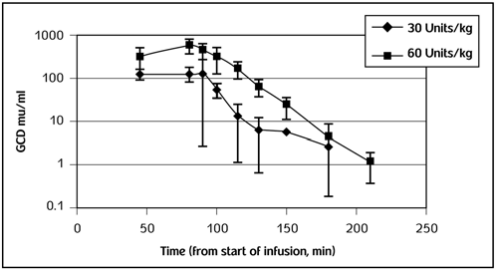
The combined pharmacokinetic profile of 5 subjects at 30 units/kg and 60 units/kg. Blood samples were collected prior to dosing (0) and at predetermined time points after start of infusion. Plasma was analyzed for prGCD concentration using a validated immunoassay. Error bars represent standard deviation. Plasma analysis data were analyzed by Noncompartmental pharmacokinetic analyses (NCA) using WinNonlin® software.

Comparison of prGCD concentration versus time for males versus females at each dose level revealed no clear difference in exposure between genders. Combining prGCD concentration for males and females and plotting mean concentration versus time for all three doses indicate dose-dependence in exposure to prGCD. The pharmacokinetic profile, combined for 5 patients at 30 units/kg and 60 units/kg is presented in Figure 1. All AUC calculations were based on the time interval from start of infusion to the last measurable plasma concentration (AUC_last_). Inspection of the NCA results also confirmed no obvious difference between males and females for C_max_ and AUC_last_ for these small group sizes. A mean half-life (t½) of approximately 15 min (range: 8–32 min) was determined and is based on all subjects in the 30 and 60 units/kg dose groups. At 30 and 60 units/kg, individual CL values ranged from 0.8 to 3.4 mL/min/kg. Volume of distribution (Vss) estimates ranged from 34 to 94 mL/kg at 30 and 60 units/kg, which is consistent with the size of the human plasma compartment.

## Discussion

The primary objective of this Phase I clinical trial was to determine the safety of recombinant plant cell expressed glucocerebrosidase (prGCD) in healthy volunteers. The safety results confirmed that there was no clinical or laboratory evidence of any significant innate or humoral immune reactions of this investigational drug with any clinically significant adverse events. prGCD administered IV once a week up to 60 mg/kg was shown to be safe and non immunogenic. This data was strongly supported by pre-clinical toxicological studies in animals including primates, performed with prGCD. Based on these results, the US Food and Drug Administration (FDA) allowed prGCD to proceed to a Phase 3 pivotal clinical trial, which is currently ongoing. An interesting finding in this study comes from the pharmacokinetic results, wherein the half life of prGCD was prolonged relative to that of Cerezyme® (Genzyme Corporation, MA, USA), the commercially available enzyme, reported as 3.6–10.4 min [16](Genzyme website). While these data are based on a relatively small number of human subjects, these findings have been corroborated in the primate studies as well, and therefore probably reflect a truly prolonged half life of the prGCD enzyme. At this stage, it is impossible to predict whether this longer circulation time in the blood would have any clinical advantage based on a longer time of exposure to the mannose receptors on the macrophages. Given the great similarity between the prGCD and Cerezyme® proteins, already reported at the level of their 3-D structure [14], and based on the increased uptake of prGCD by the tissue macrophages [14], it will be interesting to compare the clinical outcome of prGCD treatment with the reported outcome Cerezyme® treatment.

Development of antibodies to the current commercially available recombinant human glucocerebrosidase, expressed in CHO cells, has been reported in approximately 15% of the treated patients tested and antibody formation was reported following 3–12 months of treatment [17], [18], [19]. Considering the relatively small group size and the short duration of treatment (total of 4 weeks) in the human study, the lack of of antibodies to prGCD formation is not surprising. This may also attributed to the high homology with the endogenous protein [14]. The differences between the reactions of the primates and human subjects has also to do with the differences in the amount of prGCD given and the frequency and duration of dosing (up to 27.8 mg/ml for 9 months in primates and only up to 1.8 mg/ml in humans over a 4 week period). The formation of antibodies to prGCD in Gaucher patients will be further evaluated in the Phase 3 clinical study.

There is a public concern regarding the high cost of the recombinant GCD enzyme approved for Gaucher's disease [3], [12], [13]. The plant cell system used for the expression of prGCD has the potential to be highly advantageous due to the safe and cost effective production of therapeutic recombinant glycoproteins that are not suitable for production in bacterial hosts, and are currently being expressed in costly mammalian expression systems. prGCD is produced in carrot cells, naturally possesses terminal Mannose residues and therefore, prGCD production does not require costly enzymatic deglycosylation as would be required for the production of recombinant GCD in mammalian cells [14]. Furthermore, it provides high batch-to-batch reproducibility of its glycan structures, which represents another important advantage of plant cells. An additional advantage over mammalian cells is the fact they do not involve mammalian-derived components in the manufacturing process, making the purified biopharmaceutical products less expensive and potentially safer [3], [20], [21].

It remains to be determined in larger scale clinical studies, as required in any new protein drug development process, whether plant-derived biopharmaceutical glycoproteins are associated with any excessive immunogenicity effects or excessive neutralizing antibody formation, beyond the standard rate seen for recombinant therapeutic proteins, which may limit their use. However, the current regulatory viewpoint, based on data accumulated in a number of clinical trials involving different plant-expressed proteins, is quite promising in this regard [15], [21]. The clinical and preclinical data presented herein and reported previously [14], demonstrated that there were no obvious treatment-related adverse reactions or clinical findings, indicating the potential safety of plant cell-expressed GCD.

Currently, several biotechnology companies have used plant biotechnology to produce protein pharmaceuticals, such as glucocerebrosidase to treat Gaucher disease in our case, lipase to treat cystic fibrosis, alpha-interferon, lactoferrin, and others, which are under evaluation in human studies [15]. The value and impact of plant biotechnology on global health should not be dismissed. Protein drugs, although quite costly, are widely used in the developed world, but economic barriers make most of these new biotechnology products inaccessible to the general public, but the very wealthiest inhabitants of the developing world. It is likely that another opportunity for plant-made biopharmaceuticals will take place in the field of new versions of biopharmaceutical products or “biosimilar” versions of existing protein drugs. This is especially true as patents on current drugs expire [22] or in those cases where the patents do not protect the expression method of such proteins in plants. The ongoing clinical trials evaluating these different plant biotechnology produced protein-based pharmaceuticals will enable to determine the merit of using these novel and highly promising technologies.

## Supporting Information

Checklist S1 CONSORT Checklist
(0.06 MB DOC)

Protocol S1 Trial Protocol
(1.72 MB DOC)
