# Supplementary material for: A Plant-Derived Recombinant Human Glucocerebrosidase Enzyme—A Preclinical and Phase I Investigation
Source: PLoS One. 2009 Mar 11;4(3):e4792. doi: 10.1371/journal.pone.0004792 (PMC2652073; doi:10.1371/journal.pone.0004792)
Supplement: Protocol S1 — Trial Protocol (1.72 MB DOC) [file pone.0004792.s002.doc]

# Clinical Protocol

**PROTOCOL #P-01-2005**

**A Phase I, Non-Randomized, Open Label, Single Dose-Escalation Safety Study of Recombinant Human Glucocerebrosidase (prGCD) in Healthy Volunteers**

#

#

#
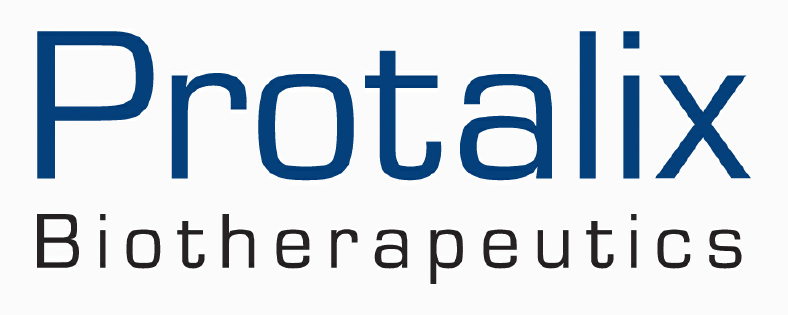


#

**UPDATED FOR – 30 August 2005**

**CONFIDENTIAL** **DO NOT COPY**

**This investigational protocol is the property of Protalix, Ltd and contains confidential information for use by the principal investigators and their designated representatives participating in this clinical investigation. It should be held confidential and maintained in a secure location. It should not be copied or made available for review by any unauthorized person or firm.**

**PROTOCOL #P-01-2005**

**A Phase I, Non-Randomized, Open Label, Single Dose-Escalation Safety Study of Recombinant Human Glucocerebrosidase (prGCD) in Healthy Volunteers**

| **Sponsor:** | Protalix Ltd.  2 Snunit St.  Science Park  POB 455  Carmiel 20100, Israel  Ph: 972-4-988-9488  Fax: 972-4-988-9489 |
| --- | --- |
|  |  |
| **Sponsor Contact:** | Dr. Einat Almon  Telephone: 972-4-9889488/ext.137  Fax: 972-4-9889489 |
|  |  |
| **Clinical Monitor:** | Quintiles Israel Ltd.  1 Hamachshev st.  Poleg Industrial Park  Netanya 42504, Israel  Telephone: 972-9-8634040  Fax: 972-9-8634044 |
|  |  |
| **Date of Protocol:** | June 28, 2005 |
|  |  |
|  |  |
|  |  |
|  |  |
|  |  |
|  |  |
|  |  |
| **Proprietary Notice:** | The concepts and information contained herein are considered proprietary and shall not be disclosed in whole or in part without the expressed written consent of Protalix Ltd. |

**PROTOCOL APPROVAL**

**Protocol Number: P-01-2005**

**Title: A Phase I, Non-Randomized, Open Label, Single Dose-Escalation Safety Study of Recombinant Human Glucocerebrosidase (prGCD) in Healthy Volunteers**

**Investigator’s Statement and Signature**

I have read and understand this protocol and concur with the study design. I agree to participate as a Principal Investigator and to follow the protocol as outlined.

**_____________________________ _____________________**

Prof. Eithan Galun

Principal Investigator Date

(Signature)

**SPONSOR SIGNATURE PAGE**

The undersigned are the persons authorized to sign the protocol on behalf of the sponsor.

Date:

Dr. Einat Almon

Senior Director Product Development

**PROTOCOL SYNOPSIS**

**Protocol Number:** P-01-2005

**Title:** A Phase I, Non-Randomized, Open Label, Single Dose, Dose-Escalation Safety Study of Recombinant Human Glucocerebrosidase (prGCD) in Healthy Volunteers

**Study Phase:** 1

**Study Site:** Jerusalem Phase I unit, Hadassah Medical Organization, Jerusalem, Israel

**Test Material:** human prGCD 15 units/kg; 30 units/kg; 60 units/kg and vehicle

**Dose:** human prGCD will be administered as an intravenous infusion (IV) delivered over 1.5 hour. The rate of infusion is to be 135 mL/1.5 hr (1.5ml/minute). The infusion rate will be the same over 90 minutes for all doses. All subjects will receive all doses in a sequential manner starting with vehicle. Subjects will be closely monitored for 2 hours from the time of the initiation of the infusion.

Vehicle will be given at the baseline visit. The initial human prGCD dose of 15 units/kg will be given at Day 8; 30 units/kg dose will be administered on Day 15, and 60 units/kg dose on Day 22.

**Route of Administration:** Intravenous (IV)

**Controls:** Each subject will receive vehicle and thereby will be his/hers own control.

**Objectives:**

**Primary**

To evaluate the safety of three escalating doses of human Glucocerebrosidase (prGCD) intravenously administered once a week in healthy volunteers.

**Secondary**

To evaluate the pharmacokinetic profile of prGCD after IV administration in healthy volunteers

**Design:** This is a phase I, non-randomized, open label, safety study of three escalating single doses of human prGCD administered as an intravenous infusion (IV) in healthy volunteers.

Eligible subjects will receive the vehicle on Day 1 followed by human prGCD on Day 8 (15 units/kg), Day 15 (30 units/kg), and Day 22 (60 units/kg). At each visit, blood samples for clinical chemistries and CBC with a differential count, will be collected prior to dosing and at 24h post the initiation of the infusion. Blood samples for PK analysis will be collected at 0, 5, 45, 80, and 90 minutes during the infusion and 100, 115, 130, 150, 180, 210 minutes, and 24 hours post-infusion. Subjects will be discharged from the clinic after 8 hr on the day of the infusion and will arrive again 24 hr from the initiation time of infusion for blood draw and additional safety assessment.. A follow-up visit will occur on Day 29.

**Blinding:** Open-label study

**Sample Size:** A total of 6 healthy volunteers will participate

**Method of Subject Assignment:** Open label

**Primary Outcome Measures:** Safety as measured by adverse events, changes in vital signs, physical examination and laboratory test results.

**Secondary Outcome Measures:**

- Pharmacokinetic parameters
- Immunological profile including: IgE, anti human prGCD antibodies, eosinophils and proteinuria

**Plan for Obtaining Adverse Events:** All volunteered, elicited and observed adverse events (AEs) will be documented.

**Plan for Data Analyses:** Safety parameters, as well as changes from baseline, will be examined and summarized for descriptive purposes. Safety will also be assessed through the recording of adverse events. Adverse events will be classified by body system using the MedDRA classification, and tabulated based on the incidence, severity, and causality to study treatment.

TABLE OF CONTENTS

[LIST OF ABBREVIATIONS 9](#__RefHeading___Toc102708696)

[1. INTRODUCTION 10](#__RefHeading___Toc102708697)

[1.1. Rationale 10](#__RefHeading___Toc102708698)

[1.2. Summary of non clinical and clinical studies 10](#__RefHeading___Toc102708699)

[2. OBJECTIVES 11](#__RefHeading___Toc102708700)

[2.1. Primary 11](#__RefHeading___Toc102708701)

[2.2. Secondary 11](#__RefHeading___Toc102708702)

[3. STUDY DESIGN 11](#__RefHeading___Toc102708703)

[3.1. Indication 12](#__RefHeading___Toc102708704)

[3.2. Blinding 12](#__RefHeading___Toc102708705)

[3.3. Controls 12](#__RefHeading___Toc102708706)

[3.4. Randomization 12](#__RefHeading___Toc102708707)

[3.5. Dose Rationale 12](#__RefHeading___Toc102708708)

[3.6. Stopping Criteria 13](#__RefHeading___Toc102708709)

[3.7. Study Flow Chart 13](#__RefHeading___Toc102708710)

[4. SUBJECT POPULATION 13](#__RefHeading___Toc102708711)

[4.1. Inclusion Criteria 13](#__RefHeading___Toc102708712)

[4.2. Exclusion Criteria 13](#__RefHeading___Toc102708713)

[5. STUDY PROCEDURES AND VISIT SCHEDULE 14](#__RefHeading___Toc102708714)

[5.1. Pre-Screening Visit (Day -1) 14](#__RefHeading___Toc102708715)

[5.2. Screening Visit (Day 0) 14](#__RefHeading___Toc102708716)

[5.3. Baseline Visit (Visit 1, Day 1, Treatment visit) Baseline Visit can be done ± 1 day from planned visit. 14](#__RefHeading___Toc102708717)

[5.4. Visits 2-2a, 3-3a and 4-4a (Treatment Visits, Day 8-9, Day 15-16, and Day 22-23), visit 2,3 and 4 can be done ± 1 day from planned visit. 15](#__RefHeading___Toc102708718)

[5.5. Visit 5 (Follow-up Visit, Day 29) 16](#__RefHeading___Toc102708719)

[5.6. Observations and Measurements 16](#__RefHeading___Toc102708720)

[5.6.1. Blood Tests. 16](#__RefHeading___Toc102708721)

[5.6.2. PK Analysis 16](#__RefHeading___Toc102708722)

[5.6.3. Adverse Events 16](#__RefHeading___Toc102708723)

[6. STUDY MEDICATION 17](#__RefHeading___Toc102708724)

[6.1. Dosage and Formulation 17](#__RefHeading___Toc102708725)

[6.1.1. Dosage 17](#__RefHeading___Toc102708726)

[6.1.2. Formulation 17](#__RefHeading___Toc102708727)

[6.2. Study Drug Administration 18](#__RefHeading___Toc102708728)

[6.3. Blinding 18](#__RefHeading___Toc102708729)

[6.4. Packaging 18](#__RefHeading___Toc102708730)

[6.5. Preparation and Labeling 19](#__RefHeading___Toc102708731)

[6.6. Storage 19](#__RefHeading___Toc102708732)

[6.7. Drug Accountability 19](#__RefHeading___Toc102708733)

[6.8. Overdosages 19](#__RefHeading___Toc102708734)

[7. ADVERSE EVENTS 20](#__RefHeading___Toc102708735)

[7.1. Definitions of Severity and Relationship to Study Medication 20](#__RefHeading___Toc102708736)

[7.1.1. Relationship to Study Medication 20](#__RefHeading___Toc102708737)

[7.1.2. Severity 20](#__RefHeading___Toc102708738)

[7.2. Serious Adverse Event Reporting 21](#__RefHeading___Toc102708739)

[7.3. Documentation of Adverse Events 21](#__RefHeading___Toc102708740)

[7.4. Current and Concomitant Medications 22](#__RefHeading___Toc102708741)

[8. SUBJECT WITHDRAWAL 22](#__RefHeading___Toc102708742)

[9. DATA ANALYSIS AND STATISTICS 23](#__RefHeading___Toc102708743)

[9.1. Study Design 23](#__RefHeading___Toc102708744)

[9.2. Primary Endpoint 23](#__RefHeading___Toc102708745)

[9.3. Secondary Endpoint 23](#__RefHeading___Toc102708746)

[9.4. Statistical Methods and Sample Size Justification 23](#__RefHeading___Toc102708747)

[9.4.1. Baseline Measurements 23](#__RefHeading___Toc102708748)

[9.4.2. Safety Assessments 23](#__RefHeading___Toc102708749)

[9.4.3. Pharmacokinetics 23](#__RefHeading___Toc102708750)

[9.4.4. Sample Size 23](#__RefHeading___Toc102708751)

[9.4.5. Definition of the Analytical Population 24](#__RefHeading___Toc102708752)

[10. Ethics and Regulatory ISSUES 24](#__RefHeading___Toc102708753)

[10.1. Compliance 24](#__RefHeading___Toc102708754)

[10.2. Institutional Review Board (IRB) Approval 25](#__RefHeading___Toc102708755)

[10.3. Protocol Amendments and Emergency Deviations 25](#__RefHeading___Toc102708756)

[10.4. Informed Consent 25](#__RefHeading___Toc102708757)

[10.5. Confidentiality 27](#__RefHeading___Toc102708758)

[11. administration 27](#__RefHeading___Toc102708759)

[11.1. Study Site Initiation 27](#__RefHeading___Toc102708760)

[11.2. Monitoring of the Study 28](#__RefHeading___Toc102708761)

[11.3. Case Report Forms and Study Records 28](#__RefHeading___Toc102708762)

[11.4. Record Retention 28](#__RefHeading___Toc102708763)

[11.5. Protocol Deviations 28](#__RefHeading___Toc102708764)

[12. Quality control and quality assurance 28](#__RefHeading___Toc102708765)

[13. Study protocol agreement form 29](#__RefHeading___Toc102708766)

[14. PUBLICATION 29](#__RefHeading___Toc102708767)

[15. REFERENCES 30](#__RefHeading___Toc102708768)

[16. APPENDIX 1: Study flow chart 31](#__RefHeading___Toc102708769)

[17. APPENDIX 2: Common Toxicity Criteria 32](#__RefHeading___Toc102708770)

LIST OF ABBREVIATIONS

| AE | Adverse Event |
| --- | --- |
| ANF | Anti Nuclear Factor |
| AST | Aspartate Aminotransferase |
| AUC | Area Under the Curve |
| CBC | Complete Blood Cell including differential count of white blood cells |
| cGMP | Current Good Manufacturing Practice |
| CHO | Chinese Hamster Ovary |
| CPK | Creatine Phosphokinase |
| CRF | Case Report Form |
| CTC | Common Toxicity Criteria |
| ECG | Electrocardiogram |
| ER | Endoplasmic Reticulum |
| ERC | Ethical Review Committee |
| ERT | Enzyme Replacement Therapy |
| FDA | Federal Drug Administration |
| GCP | Good Clinical Practice |
| GlcCer | Glucocerebroside (Glucosylceramide) |
| GLP | Good Laboratory Practice |
| HBsAg | Hepatitis B Surface Antigen |
| HCV | Hepatitis C Virus |
| HIV | Human Immunodeficiency Virus |
| ICH | International Conference on Harmonization |
| IgE | Immunoglobulin E |
| IRB | Institutional Review Board |
| IV | Intravenous |
| LDH | Lactate Dehydrogenase |
| MedDRA | Medical Dictionary of Regulatory Activities |
| MOH | Ministry of Health (Israel) |
| NDA | New Drug Application |
| PI | Principal Investigator |
| PK | Pharmacokinetics |
| pNP-Glc | para-Nitrophenyl-beta-D-Glucopyranoside |
| prGCD | Plant Cell Expressed Recombinant Human Glucocerebrosidase |
| PT | Prothrombin Time |
| PTT | Partial Thromboplastin Time |
| SAE | Serious Adverse Event |
| WFI | Water For Injection |

# INTRODUCTION

Gaucher disease, the most prevalent lysosomal storage disorder, is caused by mutations in the human glucocerebrosidase gene (GCD) leading to reduced activity of the lysosomal enzyme glucocerebrosidase and thereby to the accumulation of substrate glucocerebroside (GlcCer) in the cells of the monocyte-macrophage system. This accumulation leads to the visceral manifestations of hepatosplenomegaly, anemia and thrombocytopenia, as well as to the skeletal features and less frequently also to lung involvement.

The identification of GCD deficiency as the etiology of Gaucher disease stimulated the development of enzyme replacement therapy (ERT) as a therapeutic strategy for this disorder, which has been proven as safe and effective over the past 14 years, in over 3000 patients using either natural or recombinant purified human glucocerebrosidase derived from mammalian tissue culture production systems. Studies have shown that glycosylation plays a crucial role in glucocerebrosidase activity and uptake to target cells, and indeed, in the formulations currently used special steps of deglycosylation are required in order to generate exposed mannose residues as the terminal glycoside side chains (Bijsterbosch MK. et al. 1996; Friedman B. et al. 1999; Furbish FS. et al. 1981; Doebber T. et al. 1982).

Several approaches are currently being utilized to control and tailor protein glycosylation in plants, including specific targeting to subcellular compartments and the genetic modification of glycosylation machinery. As mention above, when human glucocerebrosidase is produced in mammalian cell culture, the glycosylation must be remodeled in order to generate an active form of the enzyme. Therefore the expression of human glucocerebrosidase in plants cells resulting in high mannose structures during production in the plant tissue culture system is of great value.

## Rationale

Human glucocerebrosidase has been used successfully for the clinical treatment of Gaucher disease for several years from both natural and recombinant sources (CHO production platform). This is the first trial to utilize a recombinant active form of lysosomal enzyme, glucocerebrosidase, (human prGCD) which is expressed and purified in a bioreactor system from transformed carrot plant root cell line.

Since the glycosylation pattern of recombinant human glucocerebrosidase must possess high mannose structures to increase uptake in target cells, the expression of human glucocerebrosidase in plant cells could be of great value. As mentioned above several approaches are utilized to control and tailor protein glycosylation in plants (Dwek RA *et al*. 2002). Protalix’s approach attempts to localize the expression to a specific compartment in the cell. For example, retaining the protein in the endoplasmic reticulum (ER) prevents plant specific modification from being carried out in the Golgi (Neuhaus JM and Rogers JC 1998; Vitale A and Galili G 2001). This approach has been adopted in developing active human prGCD.

## Summary of non clinical and clinical studies

Two GLP toxicology studies where performed and completed with the current human prGCD recombinant protein, derived from plant tissue culture bioreactor system. The first was a study in rodents, performed at Harlan Biotech Israel, In this study a single dose of: Vehicle; 1X; 5X; 10X of the clinical dose (60units/kg) were given to 24 mice. A 14-day observation period included clinical signs, body weight, detailed necropsy and gross pathological examination. In this study no obvious treatment related adverse reactions, no gross pathological findings, and no mortality were observed.

The second study was a 28 days repeat dose in non-human primates (Marmosets) preformed at Huntingdon Life Sciences Laboratory UK. Twenty-four (24) animals (4/sex/group/dose) were intravenously infused daily with 1X or 5X of the clinical dose (60units/kg) adjusted to animal body surface area The 1X clinical dose, 60U/kg, is equivalent to 1.8 mg/kg in humans which corresponds to 66 mg/m2 (using the standard human surface area conversion factor of 37). The dose of 66mg/m2 corresponds to 11 mg/kg in marmosets (using the conversion factor of 6 for marmoset monkeys). Therefore, 5 times the clinical dose is 55mg/kg in Marmoset monkeys.

In-life data were collected and analyzed. Preliminary results indicated no obvious treatment related adverse reactions, no gross pathological findings, and no mortality. Ophthalmoscopy and electrocardiography indicated no findings related to the treatment. In addition, all blood and urine tests were found to be within normal ranges for Marmosets. More detailed description of the above toxicology study can be found in the pre-clinical section in the Investigator’s Brochure.

# OBJECTIVES

## Primary

To evaluate the safety of administration of three escalating single intravenous doses of human glucocerebrosidase (prGCD) in healthy volunteers

## Secondary

To determine the pharmacokinetic profile of human prGCD after IV administration in healthy volunteers

# STUDY DESIGN

The proposed investigation will be a phase I, single-center, non-randomized, open label, safety study of three single escalating doses of human prGCD administered as an intravenous infusion (IV) to six healthy volunteers. Subjects meeting the entry criteria will be eligible to receive study drug. Urine will be collected 24 hours prior to screening to assess renal function. Seven visits are scheduled for this study: pre-screening (Day -1), Screening (Day 0), baseline (Day 1), Visit 1 (Day 8), Visit 2 (Day 15), Visit 3 (Day 22) and follow-up visit (Day 29). The study procedures for each visit are presented in the study flow chart (Appendix 1). The duration of study is 29 days.

Subjects will be monitored over 24 hours from the commencement of the infusion of each dose at the Hadassah medical center, Phase I unit. The treatment will consist of a single dose infused for 1.5 hours (1.5mL/min). The infusion rate may be adjusted according to subject symptoms and signs: slower rate (1mL/min) upon adverse effect or faster (2mL/min.) upon lack of any symptom and signs. The vital signs for each subject will be monitored during infusion. ECG will be done at baseline and at 8 hours following the infusion. Subjects will remain at the clinic for 8 hours post-infusion and will be discharged if no AEs are observed. Subjects will return to the clinic 24h post initiation of infusion for additional safety assessment. Vital signs will be recorded during the infusion and PK sampling every 30 minutes (up to 3.5 hrs), and afterward at 8 and 24hrs post infusion.

Each subject will receive a single IV dose of vehicle at the baseline visit (Day 1). Blood samples for PK will be collected prior to dosing (0) and at 5, 45, 80 and 90 minutes during the infusion and 100, 115, 130, 150, 180, 210 minutes and 24 hours from initiation of infusion. On Day 8, subjects will receive a single 15 units/kg of study drug and will have the same blood samples taken as per Day 1. On Day 15, subjects will receive a single 30 units/kg of study drug and have the blood samples taken as per Day 1. On Day 22, subjects will be administered a single dose of study drug of 60 units/kg, and have the same procedures performed as Day 1.

The Principle Investigator will review all test results and AE report 24h prior to escalation to the next dose level. The dose will be increased based on review of drug toxicity by the Principle Investigator and the Medical Director of Protalix Ltd. A follow up safety visit will take place at Day 29.

Plasma levels of human prGCD will provide a pharmacokinetic profile, including AUC_0-inf_, T_max_, C_max_ and C_min_ and will be calculated for each subject.

All PK samples of human prGCD will be analyzed at the end of the study for all subjects. This data will be used as adjunct information for a safety review, which will include a review of adverse events, changes in vital signs, physical examination and clinical laboratory test results. Unexpectedly high plasma concentrations will be reviewed for correlation to related adverse events.

This study is conducted according to FDA and European GCP guidelines.

## Indication

For the treatment of Gaucher Disease

## Blinding

Not applicable

## Controls

Not applicable

## Randomization

No randomization

## Dose Rationale

In order to enhance the healthy volunteers’ safety, all 6 subjects will undergo a staggered dose escalation starting with vehicle only, followed by low dose of 15 units/kg, continued with 30 units/kg (moderate dose) and finally with the high dose of 60 units/kg. The last 2 doses are the ones planned for phase III study. The two higher doses have been used before with different enzymatic preparations and have been shown to be safe and effective in patients with Gaucher disease.

.

## Stopping Criteria

If any subject experiences a serious adverse event, that in the judgment of the Principle Investigators and/or the Medical Director of Protalix Ltd. is life-threatening and definitely related to study medication (see Section 7.2), the study will be stopped

## Study Flow Chart

See Appendix 1

# SUBJECT POPULATION

## Inclusion Criteria

The following are the inclusion criteria:

1. Healthy male or female between 18 and 45 years of age.
2. Female subjects must agree to use a medically acceptable method of contraception at all times during the study and must have a negative serum pregnancy test at baseline and during the study period.
3. Females of child-bearing potential must be non-pregnant and not lactating and using adequate birth control such as oral contraceptives
4. Negative laboratory tests for HIV, HBsAg or HCV
5. Naïve to any previous recombinant protein therapy
6. Provide written informed consent
7. Have the ability to understand the requirements of the study and to comply with the study protocol and dosing regimen

## Exclusion Criteria

Subjects will be excluded from the study if they:

1. Have clinical evidence of any active significant disease that could potentially compromise the ability of the investigator to evaluate or interpret the effects of the study treatment on safety assessment and thus increase the risk to the subject to unacceptable levels.
2. Are pregnant or nursing.
3. Presence of any acute or chronic diseases
4. Have a history of any allergies
5. Have been exposed to long-term steroid treatment
6. Had a major operation in last 6 months
7. Have ever been exposed to any previous recombinant protein therapy
8. Have received immuno-suppressive treatment
9. Have a positive HIV, HBsAg and HCV laboratory result
10. Use any medication in cases of chronic drug administration other than vitamins or oral contraceptive (for female).
11. Have participated in another clinical trial during the previous 3 months
12. Have history of alcohol or drug abuse
13. Are considered by the Investigator to be unsuitable candidate for this study

# STUDY PROCEDURES AND VISIT SCHEDULE

## Pre-Screening Visit (Day -1), Pre-Screening Visit must be performed 24 hr before Screening visit

Urine collection 24 hours prior to screening visit to assess renal function

## Screening Visit (Day 0), Screening Visit can be done 21 day before planned visit.

1. Investigator will review all inclusion and exclusion criteria and determine subject's eligibility
2. Informed consent is signed
3. Demographics (date of birth, gender, ethnic origin)
4. Medical history (including concomitant medication use and medical conditions)
5. Complete physical examination including vital signs (blood pressure and heart rate), body weight (kg) and height (cm)
6. Chest X-ray (if not obtained within the past 6 months prior to study start), if an Chest X-ray was done within the past 6 month the film must be available for reading by the PI
7. Electrocardiogram (ECG)
8. Withdrawal of 22 mL venous blood for laboratory tests, which include,:
   1. Serum chemistries including electrolytes (K, Na, Cl, HCO_3_, Ca, and P), liver function tests (AST, ALT, LDH, AlkP, GGT, and Bilirubin), Renal function tests (BUN, and Creatinine,) CPK, Serum proteins(Albumin,Globulin), Cholesterol, and Uric Acid
   2. Complete blood cell count including white blood cells differential count (CBC), and coagulation profile (PT, PTT)
   3. ANF, ESR, C3
   4. Serology: HBsAg, HCV and HIV
   5. Serum pregnancy test
9. Subjects failing to meet the entry criteria will be consider screening failures. A screening log, containing the screening number, subject’s initials and reason(s) for failure, is to be maintained by the investigator in the study files.

## Baseline Visit (Visit 1-1a, Day 1-2, Treatment visit) Baseline Visit can be done ± 1 day from planned visit.

1. Baseline data will be collected at time 0 immediately prior to infusion of study medication and at 24h (baseline a) from initiation of infusion.
2. Body weight, vital signs and physical examination.
3. Concomitant medications
4. Electrocardiogram (ECG) (will be performed only at time 0 and 8hr post infusion.)
5. Withdrawal of 22 mL venous blood for laboratory tests, pre infusion (time 0) and 24h post infusion which include:
   1. Serum chemistries including electrolytes (K, Na, Cl, HCO_3_, Ca, and P), liver function tests (AST, ALT, LDH, AlkP, GGT, and Bilirubin), Renal function tests (BUN, and Creatinine,) CPK, Serum proteins(albumin and globulin), Cholesterol, and Uric Acid
   2. Complete blood cell count (CBC), and coagulation profile (PT, PTT)
   3. ANF, ESR, C3 (C3 test should be done both at time 0 and 24hr post infusion)
6. Urinalysis
7. 5mL PK blood for plasma sample prior to dosing
8. Prepare drug (vehicle) for infusion (see Section 6.5)
9. Administer the investigational drug (see Section 6.2) and monitor safety as described below:

| Parameters | Pre-infusion | During and Post-infusion |
| --- | --- | --- |
| Temperature | + | +^a^ |
| Blood Pressure | + | +^a^ |
| Pulse Rate | + | +^a^ |
| Respiratory Rate | + | +^a^ |

^a^ Vital signs will be measured prior to infusion and every 30 minutes during infusion and at 8, 24 hours post infusion

1. Withdrawal of 5 ml blood for plasma samples at 0,80, 210 minutes and 24 hours post-infusion.
2. Withdrawal of 5ml blood for serum for antibody detection and characterization at 24h post infusion
3. Adverse events

## Visits 2-2a, 3-3a and 4-4a (Treatment Visits, Day 8-9, Day 15-16, and Day 22-23), visit 2, 3 and 4 can be done ± 1 day from planned visit.

1. Baseline data will be collected at time 0 immediately prior to infusion of study medication and at 24h from initiation of infusion.
2. Body weight, vital sign (will be monitored during infusion) and physical examination.
3. Concomitant medications
4. Electrocardiogram (ECG) (will be performed only at time points 0 and 8 hr.)
5. Prepare drug for infusion (Section 6.5)

- Infuse 15units/kg (Visit 2)
- Infuse 30 units /kg (Visit 3)
- Infuse 60units/kg (Visit 4)

1. Concomitant medication use
2. Withdrawal of 22 mL venous blood for laboratory tests, pre infusion (time 0) and 24h post infusion which include:
3. Serum chemistries including electrolytes (K, Na, Cl, HCO3, Ca, and P), liver function tests (AST, ALT, LDH, AlkP, GGT, and Bilirubin), Renal function tests (BUN, and Creatinine,) CPK, Serum proteins(Albumin,Globulin), Cholesterol, and Uric Acid Complete blood cell count (CBC), and coagulation profile (PT, PTT)
4. ANF, ESR ,C3
5. Eosinophils, IgE
6. Urinalysis
7. Withdrawal 5ml PK blood for plasma sample prior to dosing
8. Administer the investigational drug (see Section 6.2) and monitor the same safety parameters as described in Visit 1
9. Withdrawal of 5ml blood for plasma samples at 0, 5, 45, 80 and 90 min during infusion and 100, 115, 130, 150, 180, 210 minutes and 24 hours post-infusion.
10. Withdrawal of 5ml blood for serum for antibody detection and characterization at 24h post infusion
11. Adverse events
12. Serum prGCD protein level will be checked at the end of the study for all subjects.
13. Anti human prGCD antibodies will be analyzed at the end of the study for all patients from blood serum samples taken 24hr post each infusion.

## Visit 5 (Follow-up Visit, Day 29) Visit 5 can be done ± 1 day from planned visit.

1. Adverse events
2. Concomitant medication use
3. Laboratory tests
4. Withdrawal of 22 mL venous blood for laboratory tests, which include:
   1. Serum chemistries including electrolytes (K, Na, Cl, HCO_3_, Ca, and P), liver function tests (AST, ALT, LDH, AlkP, GGT, and Bilirubin), Renal function tests (BUN, and Creatinine,) CPK, Serum proteins(Alb, Globulin), Cholesterol, and Uric Acid
   2. Complete blood cell count (CBC), and coagulation profile (PT, PTT)
   3. ANF, ESR, C3 (C3 test should be done both at time 0 and 24hr post infusion)
   4. Eosinophils, IgE
   5. Serum pregnancy test
5. Vital signs and physical examination including body weight.
6. Urinalysis
7. Withdrawal of 5ml blood for plasma for prGCD protein level detection
8. Withdrawal of 5ml blood for serum for antibody detection and characterization
9. Serum prGCD protein level will be checked at the end of the study for all subjects.
10. Anti human prGCD antibodies will be analyzed at the end of the study for all subjects.

## Observations and Measurements

### Blood Tests

Serum chemistries include electrolytes (K, Na, Cl, HCO_3_, Ca, and P), liver function tests (AST, ALT, LDH, AlkP, GGT, and Bilirubin), renal function tests (BUN, and Creatinine,) CPK, Serum proteins (Albumin and Globulin), Cholesterol, and Uric Acid.

Complete blood cell count (CBC), and coagulation profile (PT, PTT), ANF, ESR, C3, Eosinophils, and immunoglobulin including IgE.

### PK Analysis

AUC_0-4_, T_max_, C_max_ and C_min_ will be calculated for each subject using non-compartmental methods.

### Adverse Events

All adverse events (AEs) observed by the Principle Investigator or reported by the patient will be documented with respect to onset, severity, relationship to study treatment and resolution. Grading of severity will be performed according to the Common Toxicity Criteria (CTC) version 2.0 April 1999 (Appendix 2)

# STUDY MEDICATION

## Dosage and Formulation

### Dosage

Four treatment regimens will be evaluated in this study.

- Vehicle
- prGCD 15 units/kg body weight
- prGCD 30 units/kg body weight
- prGCD 60 units/kg body weight

A unit is defined as the amount of enzyme that catalyzes the hydrolysis of 1 micromole of the synthetic substrate para-nitrophenyl-beta-D-glucopyranoside (pNP-Glc) per minute at 37^o^C. Human prGCD vials are stored lyophilized at 4^o^C. The human prGCD final concentration is 40 unit/ml after reconstitution with WFI (or 0.9% NaCl). The final concentration and administration volumes are supplied in 200unit/vial.

### Formulation

Human prGCD is a purified recombinant, plant cells-expressed glucocerebrosidase, which is described in detail in the Investigator Brochure.

Each vial contains the following lyophilized contents:

200U of human prGCD

195 mg mannitol

35 mg sodium citrate

0.53 mg polysorbate 80, NF

The accuracy of preparation of the supplied formulations is the responsibility of the Sponsor.

## Study Drug Administration

Human prGCD will be administered as an intravenous infusion (IV) delivered over 1.5 hours. The rate of infusion is to be 135 mL/1.5 hrs (1.5ml/minute). The infusion rate will be the same over 90 minutes for all doses. The infusion rate may be adjusted according to subject symptoms and signs: slower rate (1mL/min) upon adverse effect or faster (2mL/min.) upon lack of any symptom and signs. Subjects will be monitored for 24 hours from the commencement of the infusion of each dose at the Hadassah medical center, Phase I unit.

## Blinding

Not applicable

## Packaging

Lyophilized drug powder is stored in 20 ml low extractable borosilicate glass bottles (Wheaton Science Product, USA or Bunder Glass, Germany). Lyophilization stoppers (Wheaton Science Product, USA) composed of two-leg grey butyl rubber are sealed with Tear-Cap® Aluminum seals (Wheaton Science Product, USA).

## Preparation and Labeling

Reconstitution with 5.1mL of Sterile Water for Injection (WFI) yields a final volume of 5.1 mL human prGCD (40 units/mL), which provides a withdrawal volume of 5.0 mL (200 enzyme units). The solution must be mixed well until clear. The required amount of enzyme units will be adjusted with normal saline (0.9%) up to 135ml/infusion.

In case a subject will weigh over 90 kg at dosing of 60 units/kg infusion volume will be increased to 150mL accordingly.

Vehicle preparation includes reconstitution as described herein above for the human prGCD, where the number of vials containing vehicle formulation is equivalent to the number of vials that are required for administering 60U/kg for each subject.

prGCD

Enzyme 6mg, 200 units

195mg Mannitol

35mg Sodium Citrate

0.53mg Polysorbate 80 N.F.

Batch # PLX-GC-XXX-XXXX Date_____

Protocol # P-01-2005

For Clinical use only

Storage 2-8^o^C


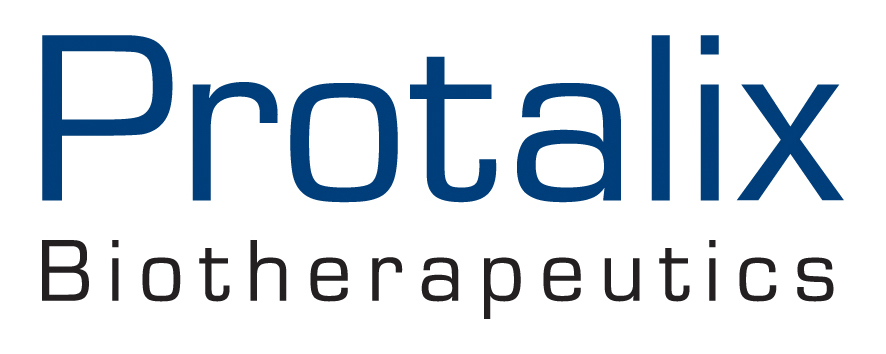


## Storage

The product is stored at 2-8°C (36-46°F).

## Drug Accountability

Protalix will provide drug accountability forms to assist the investigator in maintaining current and accurate inventory records covering receipt, dispensing, and the return of investigational drug supplies. When a shipment is received, the investigator or pharmacist will verify the quantities received and return the acknowledgment to the study monitor or designee. The investigational drug accountability record includes the study identification of the person to whom the drug is dispensed, the quantity and the date of dispensing and any returned or unused drug. This record is in addition to any drug accountability information recorded on the Case Report Form (CRF). These records will be readily available for inspection by a monitor or Protalix audits and are open to regulatory authority inspection at any time.

When the supplies are returned to Protalix, the investigator or Phase I unit site manger signs the investigational drug return log to verify that all unused or partially used supplies have been returned and that no study supplies remain in the investigator’s possession. One copy of all inventory records and the return statement are retained by the investigator for the study files.

At study termination, unless otherwise specified, all unused drug supplies including partially used containers and the study drug accountability log should be returned to Protalix, Ltd.

## Overdosages

No over-dosage to currently approved human glucocerebrosidase have been reported with either the placental derived or the recombinant glucocerebrosidase enzymes

# ADVERSE EVENTS

An adverse event is an undesirable or unintentional event that occurs during the usage of the study drug, whether or not it is related to the drug; this includes clinically significant changes in laboratory values. Regardless of the severity or relationship to the investigational drug, all adverse events occurring during the study period are to be recorded in the subject's CRFs. The phrase "associated with the use of the drug" is defined as a reasonable possibility that the event may have been caused by the drug under investigation.

## Definitions of Severity and Relationship to Study Medication

### Relationship to Study Medication

The following definitions should be used to assess the relationship between an adverse event and the study drug.

Unrelated: The event is clearly related to other factors, such as a subject's clinical state, therapeutic interventions or concomitant medications.

Remotely Related: The event was most likely produced by other factors, such as a subject's clinical state, therapeutic interventions or concomitant medications, and does not follow a known response pattern to the study drug.

Possibly Related: The event has a reasonable temporal relationship to the study drug administration and follows a known response pattern to the study drug. However, a potential alternate etiology may be responsible for the event. The effect of drug withdrawal is unclear. Rechallenge information is unclear or lacking.

Probably Related: The event follows a reasonable temporal sequence from the time of drug administration, and follows a known response pattern to the study drug and cannot be reasonably explained by other factors. There is a reasonable response to withdrawal of the drug. Rechallenge information is not available or advisable.

Related: The event follows a temporal sequence from the time of drug administration and follows a known response pattern to the study drug. It either occurs immediately following the study drug administration, improves on stopping the drug, or reappears on repeat exposure.

### Severity

Mild Adverse Events:

A mild adverse event is one where the symptoms are barely noticeable to the subject. It does not influence performance or prevent the subject from carrying on with normal life activities.

Moderate Adverse Events:

A moderate adverse event is one where the symptoms make a subject uncomfortable and cause some impairment to normal life activities. Treatment for symptom(s) may be required.

Severe Adverse Events:

A severe adverse event is one where the symptoms cause severe discomfort to the subject and severely limit the subject's normal daily activities. Treatment for symptom(s) is given. Note that serious and severe are not synonymous. A serious adverse event must fulfill the requirements listed in the definition below.

The Common Toxicity Criteria will be used for grading the severity of each AE (Appendix 2)

## Serious Adverse Event Reporting

A serious adverse event (SAE) is any event that suggests a significant hazard, contraindication, side effect, or precaution. An SAE is defined as any untoward medical occurrence that at any dose:

- - - Results in death
    - Is life-threatening
    - Requires hospitalization or prolongation of existing hospitalization
    - Results in persistent or significant disability or incapacity
    - Is a congenital anomaly, cancer, overdose or birth defect

Life Threatening is defined as an event where a subject is at risk of death at the time of the event. It does not refer to an event that hypothetically might have caused death if it was more severe. Disability is defined as a substantial disruption of a person's ability to conduct normal life functions. An unexpected adverse event is one that has not been previously observed, or one that is of a specificity or severity not consistent with the current investigator brochure.

Any serious adverse event should be reported to the Sponsor's Monitor by telephone or fax within 24 hours of the event. The call should be followed-up with a written report within five days. Full details of the event, any sequels and an assessment of the relationship to the study drug, must be provided in the report. Any serious adverse events must also be reported to the Institutional Review Board (IRB) within 48 hours and a copy of this report must be sent to the Sponsor's Monitor. Cases of death must be reported immediately to the Hospital Director. Protalix will notify the appropriate regulatory authorities as required.

In the event of an SAE, the Medical Director must be contacted immediately by calling the following telephone # within 24 hours of disclosure of the incident:

Telephone: 972-2-6795537; 972-54-5728284

All SAEs must be reported to the IRB. The investigator will promptly supply all information identified and requested by the sponsor and its contracted monitor regarding the SAE.

## Documentation of Adverse Events

All adverse events must be recorded in the CRFs. When an adverse event occurs, the following information and assessments should be recorded in the adverse event section of the CRF:

1. The symptoms, signs, abnormal laboratory finding or a diagnosis of the event.
2. The date and time of onset of the event, using the 24 hour clock, where midnight is 00:00 and noon is 12:00.
3. The adverse event severity, using the criteria outlined above.
4. The relationship of the event to the study drug as outlined above.
5. The description of any action taken regarding study drug disposition.
6. Any required therapy, medication, treatment or diagnostic procedure.

Follow-up of a subject should be conducted until resolution of the adverse event.

The investigator is responsible for the appropriate medical management of all adverse events.

The condition of each study subject will be monitored throughout the study by the treatment clinician or designee. At each visit, symptoms and signs will be observed by the treatment clinician (or designee) or volunteered by the subject. In addition, signs and symptoms that may have occurred since the previous visit will be elicited by asking open or indirect questions, such as “How have you felt since your last visit?”

All AEs, whether observed by the examination clinician, elicited from or volunteered by the subject, will be recorded on the CRF. Data will include start and end dates, investigator-specified severity and relationship to study medication, and action taken with respect to the study medication. Whether the event resulted in death, required (or prolonged) hospitalization resulted in persistent or significant disability/incapacity, required intervention to prevent any of those outcomes, and/or was reported to the sponsor as serious will also be recorded.

## Current and Concomitant Medications

The name and duration of treatment of all medications taken at the time of the inclusion or during the study will be recorded on the CRF. Dosage, route of administration and indication will also be specified.

# SUBJECT WITHDRAWAL

Subjects will be discontinued from the study prematurely if:

1. An intercurrent illness occurs which precludes further treatment.
2. A serious adverse event occurs, which in the judgment of the principle investigator, managing physicians, and/or sponsor, is probably related to the study drug and cannot be acceptably managed.
3. The subject requests to be withdrawn from the study.
4. The principal investigator decides that it is in the subject's best interest.
5. The subject is noncompliant with the protocol.
6. The sponsor decides to discontinue the study.
7. Subjects who participates in another investigational drug trial

All subjects prematurely discontinued from the trial, regardless of cause, will have follow up visits according to the study schedule, for safety. The reason(s) for withdrawal will be recorded in the CRF by the investigator and the occurrence reported to the medical monitor.

# DATA ANALYSIS AND STATISTICS

## Study Design

This is a Phase I, non-randomized, open label, dose escalating, safety study of human prGCD in healthy volunteers.

## Primary Endpoint

The primary endpoints will be the safety parameters at the end of the study, compared to baseline measurements.

The safety parameters for this study will include:

- General infusion related toxicity
- Physical examination including vital signs and body weight
- Clinical laboratory tests
- Adverse events

## Secondary Endpoint

The secondary endpoint is to determine the pharmacokinetic profile of human prGCD.

If there are sufficient numbers of measurable plasma levels of prGCD to provide a pharmacokinetic profile, AUC_0-inf_, T_max_, C_max_ and C_min_ will be calculated for each subject using non-compartmental methods. If there are few or no concentrations of prGCD above the limit of detection for the assay, the data will be tabulated.

## Statistical Methods and Sample Size Justification

### Baseline Measurements

Baseline characteristics of subjects will be summarized by descriptive statistics.

### Safety Assessments

Safety parameters at each follow-up assessment, as well as changes from baseline, will be examined and summarized for descriptive purposes. Safety will also be assessed through the recording of adverse events. Adverse events will be classified by body system using the MedDRA classification, and tabulated based on the incidence, severity, and causality to study treatment.

### Pharmacokinetics

A pharmacokinetic profile, AUC_0-t_, T_max_, C_max_, C_min_ and T_½_ will be calculated for each subject.

### Sample Size

Since this trial is a Phase I study, no formal power or sample size calculations were made. The data collected will be used to estimate possible effect size and variability of measures.

In general, all the analyses described above are considered exploratory and descriptive in nature and are designed to give some insight into the relationship between the treatment and potential outcomes.

All computations will be performed using SAS version 9.1

### Definition of the Analytical Population

Safety population is defined as subjects who received at least one dose of study medication.

# Ethics and Regulatory ISSUES

## Compliance

The study will be carried out in accordance with accepted international standards that meet Good Clinical Practice (GCP) regulations. These standards are drawn from the following guidelines:

- ICH Guideline for Good Clinical Practice, 1 May 1996, amended September 1997.
- Declaration of Helsinki (as amended in Edinburgh, October 2000), concerning medical research on humans.

The investigator(s) will ensure that this study is conducted in full conformity with the principles of the “Declaration of Helsinki” and with the laws and regulations of the state of Israel, or whichever affords the greater protection to the individual. It is the responsibility of the investigator to obtain informed consent in written form (according to local legal requirements) from each subject participating in this study. All subjects are to be informed of the aims, methods, anticipated benefits, potential hazards and confidentiality of data. Candidates will also be told that they are free to refuse participation at any time.

This study will be conducted according to Good Clinical Practice, Good Laboratory Practice, the Declaration of Helsinki, US 21 CFR Part 50 – Protection of Human Subjects, and Part 56 – Institutional Review Board.

## Institutional Review Board (IRB) Approval

The principal investigator will provide the IRB with all appropriate material, including the protocol, informed consent, investigator brochure, and any advertising materials. The study will not be initiated until the IRB provides written approval of the aforementioned documents and until approval documents have been obtained by the principal investigator and copies received by Protalix Ltd. Appropriate reports on the progress of this study by the principal investigator will be made to the IRB and Protalix Ltd. in accordance with the applicable government regulations and in agreement with policy established by the sponsor.

This study must be approved by an IRB or other ethical approval body prior to subject enrollment. The governing IRB must comply with all the requirements set forth in the ICH guidelines. The IRB is responsible for the initial approval and continuing review of the proposed clinical study. The investigator must retain a copy of the approval letter that contains the study number, protocol title and identification of all documents approved. A copy of the letter must be sent to Protalix prior to drug shipment to the investigator.

The investigator agrees to promptly report to the IRB all unanticipated problems involving risks to human subjects or others.

A final report should be submitted to the IRB and Protalix within three months after completion or termination of the study. The investigator must maintain an accurate and complete record of all submissions made to the IRB, including a list of all reports and documents submitted.

## Protocol Amendments and Emergency Deviations

The investigator also agrees not to make any changes in the study protocol or its conduct, except when necessary to prevent immediate hazards to human life. Changes in protocol may be made only by written amendment by Protalix’s project director. Protocol amendments must be approved by the IRB before implementation. Documentation of IRB approval for such amendments must be kept on file by the investigator and forwarded to Protalix Ltd.

## Informed Consent

ICH guidelines for informing research participants are outlined below. The consent form must be written in a language that the subject understands.

Basic Elements of Informed Consent

The following are the basic elements of informed consent with which each subject should be provided:

1. A statement that the study involves research, an explanation of the purposes of the research, the expected duration of the subject’s participation, a description of the procedures to be followed and identification of any procedures that are experimental.
2. A description of any reasonably foreseeable risks or discomforts to the subject.
3. A description of any benefits to the subject or to others that may reasonably be expected from the research.
4. A statement describing the extent, if any, to which confidentiality of records identifying the subject will be maintained and that notes the possibility that the Israeli MOH (Ministry of Health) or Protalix or designee may inspect the records.
5. For research involving more than the minimal risk, an explanation as to whether any compensation or medical treatments are available if injury occurs, the extent of available treatment or compensation or where this information may be obtained.
6. An explanation of whom to contact for answers to pertinent questions about the research and the research subject’s rights and whom to contact in the event of a research-related injury.
7. A statement that participation is voluntary, that refusal to participate will involve no penalty or loss of benefits to which the subject is otherwise entitled, and that the subject may discontinue participation at any time without penalty or loss of benefits to which the subject is normally entitled.

Additional Elements of Informed Consent

When appropriate, each subject will be provided with one or more of the additional elements of informed consent:

1. A statement that the particular treatment or procedure may involve risks to the subject (or the embryo or fetus if the subject is, or may become, pregnant) that are currently unforeseeable.
2. Anticipated circumstances under which the subject’s participation may be terminated by the investigator without regard to the subject’s consent.
3. Any additional costs to the subject that may result from participation in the research.
4. The consequence of a subject’s decision to withdraw from the research and procedures for orderly termination of participation by the subject.
5. A statement that significant new findings developed during the course of the research, which may relate to the subject’s willingness to continue participation, will be provided to the subject.
6. The approximate number of subjects involved in the study. A subject must give his written consent to participate in the study. This consent must be witnessed and dated and retained by the investigator as part of the study records.

Additional Information and Procedures:

Protalix Ltd. must review and approve the proposed consent form prior to submission to the IRB. The proposed consent form must contain all appropriate elements of consent as outlined in this section.

The principal investigator or designee is responsible for obtaining written informed consent from potential subjects prior to entry into the study. If a subject is incompetent and unable to give consent, then consent must be obtained from the subject’s legal guardian. The investigator must keep each subject’s signed consent form on file for inspection. A copy of the signed document must be given to the subject.

A properly executed, written informed consent in compliance with Food and Drug Administration (FDA) and Good Clinical Practice (GCP) guidelines and the Declaration of Helsinki shall be obtained from each subject prior to entering the study or performing any unusual or non-routine procedure that involves a risk to the subject. A copy of the informed consent document to be used will be submitted by the investigator to IRB for review and approval prior to the start of the study. The investigator shall provide a copy of the signed informed consent to the subject, and a copy shall be maintained in the subject's medical record. Attention is directed to the basic elements that are required to be incorporated into the consent form. Additional elements of informed consent, if appropriate, must be provided to the subject.

## Confidentiality

All information provided to the investigator by xxx, Inc. or their designates including pre-clinical data, protocols, case report forms, and verbal and written information, will be kept strictly confidential and confined to the clinical personnel involved in conducting the study. All data and medical information gathered for each subject will be identified only by a unique subject study number. It is recognized that this information may be related in confidence to the IRB. In addition, no reports or information about the study or its progress will be provided to anyone not involved in the study other than to Protalix, Inc. or their designates or in confidence to the IRB, except if required by law.

# administration

## Study Site Initiation

The investigator must file the following documents with Protalix:

1. A completed, signed and dated Statement of Investigator, FDA Form FD 1572 and a curriculum vitae for each individual named on the form. A copy of the investigator’s current medical license.
2. A signed and dated Investigator Protocol Agreement.
3. A Financial Disclosure form.
4. The laboratory certification or proficiency ratings, the normal ranges for laboratory determinations required by the protocol and the curriculum vitae of the laboratory director for the laboratory listed on FDA Form FD 1572.
5. A copy of the Institutional Review Board’s (IRB) written notification of protocol approval, study-specific informed consent form and any advertisements, notices or bulletins to be used in this study in compliance with ICH guidelines.
6. The IRB/EC name, address, a list of IRB/EC members, occupations and institutional affiliations, or a statement of compliance with relevant ICH guidelines GCP compliance must be provided.

After the investigator has filed the above documents with Protalix, he/she may begin subject recruitment for the site.

## Monitoring of the Study

The investigational site will be monitored periodically to assure satisfactory subject enrollment, data recording and protocol adherence. The frequency of monitoring may vary depending on enrollment rate and the quantity of data collection. The investigator and staff are expected to cooperate with the study monitor and to provide the study monitor with all relevant study documentation. It is essential that the investigator and study coordinator set aside a sufficient amount of time for these visits to permit an adequate review of the study’s progress and of the completed Case Report Forms.

If there is a change or addition of any personnel listed on the FDA form FD 1572, a new form reflecting the change must be completed and forwarded to Protalix Ltd.

## Case Report Forms and Study Records

Study records, including subject source documents, Case Report Forms, all test results and signed informed consent forms, must be kept on file by the investigator. A signed FDA form FD 1572 and all Institutional Review Board correspondence, including notification of protocol, consent form approval, all annual reports as well as the final report to the IRB must be retained. Other documents pertaining to the conduct of the study, including laboratory normal values, laboratory certification, monitor logs, telephone reports and all written correspondence must also be kept as part of the permanent record.

If the investigator relocates, retires, or for any reason cannot keep the study records, the records may be transferred to an acceptable designee or returned to Protalix. Protalix must be notified in writing of the name and address of the person designated to retain the study records. Study records must be maintained a minimum of two years following written notification by Protalix Ltd.

As part of responsibilities assumed by participating in the study, the investigator will maintain adequate case histories for the subjects treated as part of the research under this protocol. The investigator will maintain accurate case report forms and source documentation as part of the case histories.

## Record Retention

Records will be maintained for two years following the date of a New Drug Application (NDA) approval of the drug, or for two years after the date that the FDA has been notified that all clinical investigations of the drug have been discontinued.

## Protocol Deviations

Protocol deviations will be recorded in source documents

# Quality control and quality assurance

Protalix's quality assurance management is working in compliance with the applicable cGMP guidelines (ICH 5.13.1). Meeting the requirements of the FDA "Quality System Regulations" ensures that the clinical trial and production are conducted and data are generated, documented and reported in compliance with the protocol, GCP and the applicable regulatory requirements.

All activities are documented according to cGMP and legal requirements.

# Study protocol agreement form

I, _Prof. Eithan Galun, MD, Principal Investigator, has examined Protalix Protocol P-01-2005

Entitled**:** A Phase I, Non-Randomized, Open Label, Single Dose-Escalation Safety Study of Human prGCD in Healthy Volunteers

I agree to conduct the study according to this protocol and to comply with its requirements, subject to ethical and safety consideration.

I understand that I must keep confidential the information contained in the study documents with which I have been or will be provided.

I understand that should the decision be made by the sponsor to terminate prematurely or suspend the study at any time for whatever reason, such a decision will be communicated to me in writing. Conversely, should I decide to withdraw from the execution of the study, I will communicate immediately such decision in writing to the sponsor.

| FOR THE SPONSOR  Protalix | PRINCIPAL INVESTIGATOR |
| --- | --- |
| Name: Dr. Einat Almon | Prof. Eithan Galun |
| Date: | Date: |
|  |  |
|  |  |
|  |  |
| Signature: | Signature: |

# PUBLICATION

The investigator shall have the right to publish the results of this study following a review and approval by Protalix, 45 days prior to its submission.

All proprietary information, including Protalix operations, patent applications, formulae, manufacturing processes, basic scientific data and formulation information provided to the investigator, the methodologies used in this study, as well as proprietary information obtained during the course of the study, are confidential and will remain the sole property of Protalix. The investigator agrees not to disclose any proprietary information supplied by Protalix in any way without prior written permission.

Individual subject data obtained during this study are confidential and will not be disclosed to third parties with the following exceptions:

1. When the data are needed by a subject’s personal physician or other medical personnel responsible for the subject’s welfare.
2. For data inspection and verification by Protalix or designee, or by the Institutional Review Board (IRB).

Any manuscript or abstract produced by an investigator must be provided to Protalix for review 45 days prior to submission. Individual subject identity must not be divulged in any publication.

# REFERENCES

1. Bijsterbosch MK, Donker W, van de Bilt H, van Weely S, van Berkel TJ, Aerts JM. Quantitative analysis of the targeting of mannose-terminal glucocerebrosidase: Predominant uptake by liver endothelial cells. (1996) *Eur J Biochem* **237**, 344-349
2. Friedman B, Vaddi K, Preston C, Mahon E, Cataldo JR, McPherson JM. A comparison of the pharmacological properties of carbohydrate remodeled recombinant and placental-derived β-glucocerebrosidase: Implications for clinical efficacy in treatment of Gaucher disease. (1999) *Blood* **93**, 2807-2816
3. Furbish FS, Steer CJ, Krett NL, Barranger JA. Uptake and distribution of placental glucocerebrosidase in rat hepatic cells and effects of sequential deglycosylation. (1981) *Biochim Biophys Acta* **673**, 425-434
4. Doebber T, Wu M, Bugianesi R, Ponpipom M, Furbish F, Barranger J, Brady R, Shen T. Enhanced macrophage uptake of synthetically glycosylated human placental β-glucocerebrosidase. (1982) *J. Biol. Chem.* **257**, 2193-2199
5. Dwek RA, Butters TD, Platt FM, Zitzmann N. Targeting glycosylation as a therapeutic approach. (2002) *Nat Rev Drug Discov* **1**, 65-75
6. Neuhaus JM, Rogers JC. Sorting of proteins to vacuoles in plant cells. (1998) *Plant Mol Biol* **38**, 127-144
7. Vitale A, Galili G. The endomembrane system and the problem of protein sorting. (2001) *Plant Physiol.* **125**, 115-118

#

# APPENDIX 1: Study flow chart

|  | Pre-Screening | Screening | Visit 1-  (Baseline) | Visit 1a-  (Baseline a) | Visit 2 | Visit 2a | Visit 3 | Visit 3a | Visit 4 | Visit 4a | Follow-up visit |
| --- | --- | --- | --- | --- | --- | --- | --- | --- | --- | --- | --- |
| Day | -1 | 0 | 1 | 2 | 8 | 9 | 15 | 16 | 22 | 23 | 29 |
| Informed consent |  | x |  |  |  |  |  |  |  |  |  |
| Inclusion/exclusion criteria |  | x |  |  |  |  |  |  |  |  |  |
| Demographics |  | x |  |  |  |  |  |  |  |  |  |
| Medical history & current medications |  | x |  |  |  |  |  |  |  |  |  |
| Concomitant medications |  | x | x |  | x |  | x |  | x |  | x |
| Vital Signs |  | x | x^b^ | x | x^b^ | x | x^b^ | x | x^b^ | x | x |
| Temperature |  | x | x |  | x |  | x |  | x |  | x |
| Body Weight |  | x | x |  | x |  | x |  | x |  | x |
| Physical Examination |  | x^d^ | x |  | x |  | x |  | x |  | x |
| Chest X-ray |  | x^c^ |  |  |  |  |  |  |  |  |  |
| IV infusion |  |  | x |  | x |  | x |  | x |  |  |
| AE Assessments |  |  | x | x | x | x | x | x | x | x | x |
| ECG |  | x | x |  | x |  | x |  | x |  |  |
| Laboratory tests |  |  |  |  |  |  |  |  |  |  |  |
| Serum chemistries^e^ |  | x | x | x | x | x | x | x | x | x | x |
| CBC PT, PTT, C3, ESR, ANF^e^ |  | x | x | x | x | x | x | x | x | x | x |
| Urinalysis at 0 and 24 hours post infusion | x^g^ | x | x | x | x | x | x | x | x | x | x |
| plasma prGCD protein level (PK)^f^ |  |  | x | x | x | x | x | x | x | x | x |
| Eosinophils, IgE |  | x | x | x | x | x | x | x | x | x | x |
| Serum pregnancy test |  | x |  |  |  |  |  |  |  |  | x |
| Anti human prGCD antibodies in serum |  |  |  | x |  | x |  | x |  | x | x |
| HBsAg, HCV and HIV |  | x |  |  |  |  |  |  |  |  |  |

^a^ Visit which is scheduled 24 h post each infusion visit

^b^ Vital signs (temperature, blood pressure, pulse rate, respiratory rate and cardiac rhythm) will be measured prior to infusion and every 30 minutes during infusion and at 8 and 24 hours post infusion

^c^ If not obtained within the past 6 months prior to study start and available for reading by the PI

^d^ Including height

^e^ Withdraw 22 ml whole blood pre infusion (time 0) and 24h post infusion

^f^ Withdrawal of 7 ml blood at, 0 and at 5, 45,80 and 90 minutes during the infusion and 100, 115, 130, 150, 180, 210 minutes and 24 hours from infusion initiation

^g^ Urine collection 24 hours prior to screening visit to assess renal function

# APPENDIX 2: Common Toxicity Criteria
